# Circulating BPIFB4 Levels Associate With and Influence the Abundance of Reparative Monocytes and Macrophages in Long Living Individuals

**DOI:** 10.3389/fimmu.2020.01034

**Published:** 2020-05-29

**Authors:** Elena Ciaglia, Francesco Montella, Valentina Lopardo, Pasqualina Scala, Anna Ferrario, Monica Cattaneo, Albino Carrizzo, Alberto Malovini, Paolo Madeddu, Carmine Vecchione, Annibale Alessandro Puca

**Affiliations:** ^1^Department of Medicine, Surgery and Dentistry “Scuola Medica Salernitana”, University of Salerno, Salerno, Italy; ^2^Cardiovascular Research Unit, IRCCS MultiMedica, Milan, Italy; ^3^Vascular Pathophysiology Unit - IRCCS Neuromed, Pozzilli, Italy; ^4^Laboratory of Informatics and Systems Engineering for Clinical Research, Istituti Clinici Scientifici Maugeri, Pavia, Italy; ^5^Bristol Medical School - Translational Health Sciences, Bristol Heart Institute, University of Bristol, Bristol, United Kingdom

**Keywords:** longevity, patrolling-monocytes, plasma, M2 macrophages, FACS, immunity

## Abstract

Long-Living Individuals (LLIs) delay aging and are less prone to chronic inflammatory reactions. Whether a distinct monocytes and macrophages repertoire is involved in such a characteristic remains unknown. Previous studies from our group have shown high levels of the host defense BPI Fold Containing Family B Member 4 (BPIFB4) protein in the peripheral blood of LLIs. Moreover, a polymorphic variant of the *BPIFB4* gene associated with exceptional longevity (*LAV-BPIFB4*) confers protection from cardiovascular diseases underpinned by low-grade chronic inflammation, such as atherosclerosis. We hypothesize that BPIFB4 may influence monocytes pool and macrophages skewing, shifting the balance toward an anti-inflammatory phenotype. We profiled circulating monocytes in 52 LLIs (median-age 97) and 52 healthy volunteers (median-age 55) using flow cytometry. If the frequency of total monocyte did not change, the intermediate CD14++CD16+ monocytes counts were lower in LLIs compared to control adults. Conversely, non-classical CD14+CD16++ monocyte counts, which are M2 macrophage precursors with an immunomodulatory function, were found significantly associated with the LLIs' state. In a differentiation assay, supplementation of the LLIs' plasma enhanced the capacity of monocytes, either from LLIs or controls, to acquire a paracrine M2 phenotype. A neutralizing antibody against the phosphorylation site (ser 75) of BPIFB4 blunted the M2 skewing effect of the LLIs' plasma. These data indicate that LLIs carry a peculiar anti-inflammatory myeloid profile, which is associated with and possibly sustained by high circulating levels of BPIFB4. Supplementation of recombinant BPIFB4 may represent a novel means to attenuate inflammation-related conditions typical of unhealthy aging.

**Graphical Abstract:**
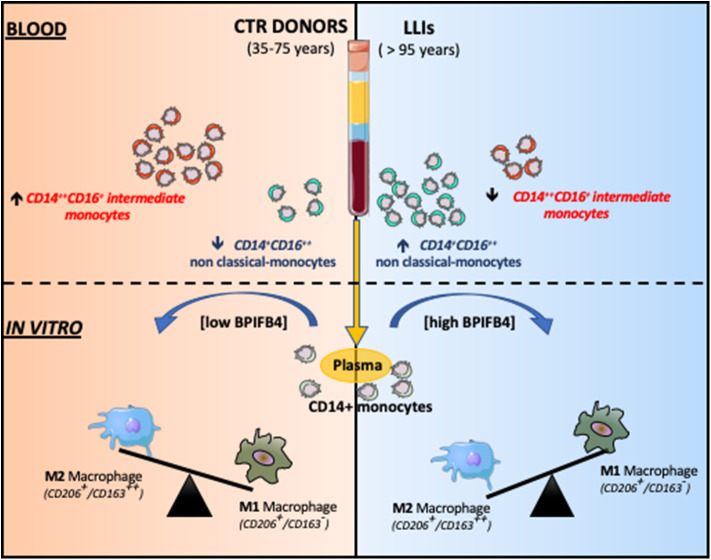
Non-classical CD14+CD16++ monocytes were found higher in LLIs compared to donors. Monocyte-derived macrophages, in presence of BPIFB4 enriched-LLIs' plasma, displayed a better tendency to acquire an anti-inflammatory M2 phenotype.

## Results and Discussion

In industrialized countries, lifespan averages 78 for males and 83 for females (1), but some exceptional individuals delay aging and live much longer than the rest of the population. Long Living Individuals (LLIs) represent a model of positive biology and an exceptional resource to study and find a way to improve general public health. Research of prolonged healthy aging could lead to important inference on mechanisms that protect from age-related diseases (1). In turn, emerging druggable targets may become blockbuster treatments capable of preventing or even overpowering unhealthy aging.

Chronic inflammation is linked with shorter life expectancy (2) and with diseases that reduce the quality of life of elderly people (*termed inflammaging*) (3). Several markers have been proposed to track systemic and vascular inflammation (4), but none has been developed yet to shed light on the opposite aspect of that subject.

We posit that the peripheral blood of LLIs may hold valuable biomarkers associated with an enduring performance of the immune system. Circulating factors unique to LLIs may also be directly involved in maintaining a proper balance between M1 (pro-inflammatory) and M2 (anti-inflammatory) macrophage phenotypes.

The bactericidal/permeability-increasing fold-containing-family-B-member-4 (BPIFB4) is one of the most abundant proteins in respiratory secretions, being highly concentrated in the upper airways and proximal trachea. BPIFB4 expression is highly responsive to airborne pathogens and participates in host protection through antimicrobial, surfactant, and immunomodulatory properties (5, 6). Of note, circulating BPIFB4 levels are constitutively increased in healthy LLIs as compared to frail ones and young controls (7). Moreover, carriers of the longevity-associated variant (LAV) have extremely prolonged life expectancy and show higher circulating BPIFB4 levels as compared with carrier of the wild-type haplotype (8). Gene therapy with *LAV-BPIFB4* was able to improve post-ischemic revascularization and endothelial function (9), and to block the atherosclerotic process in ApoE^−/−^ mice. Moreover, in two patient cohorts, circulating BPIFB4 levels were found to be correlated with less carotid stenosis and intima-media thickness (IMT) (8). The study on ApoE^−/−^ mice also revealed that LAV-BPIFB4 treatment determined an increased abundance of CXCR4+Ly6C^high^ precursor monocytes in bone marrow and spleen, the two major tissue reservoirs of monocytes available to mobilize toward injured tissues in periphery. Furthermore, LAV-BPIFB4 overexpression conferred the animals with a pro-resolving M2 macrophages profile. Similarly, *in vitro* exposure of human monocytes from atherosclerotic patients to the LAV-BPIFB4 recombinant protein caused a switch toward the M2 phenotype (8).

We then hypothesize that high circulating levels of BPIFB4 associate with and are responsible for monocytes redistribution and macrophages polarization in LLIs. To this aim, we have studied a group of 52 LLIs (median age 97, range 95–99) from the exceptional longevity cohort resident in Cilento, a rural area of Southern Italy, and compared their monocyte profile with that of two different groups of adults (35–45 years, *n* = 18) and elderly controls (65–75 years, *n* = 24) from the same region.

**Figure 1:**
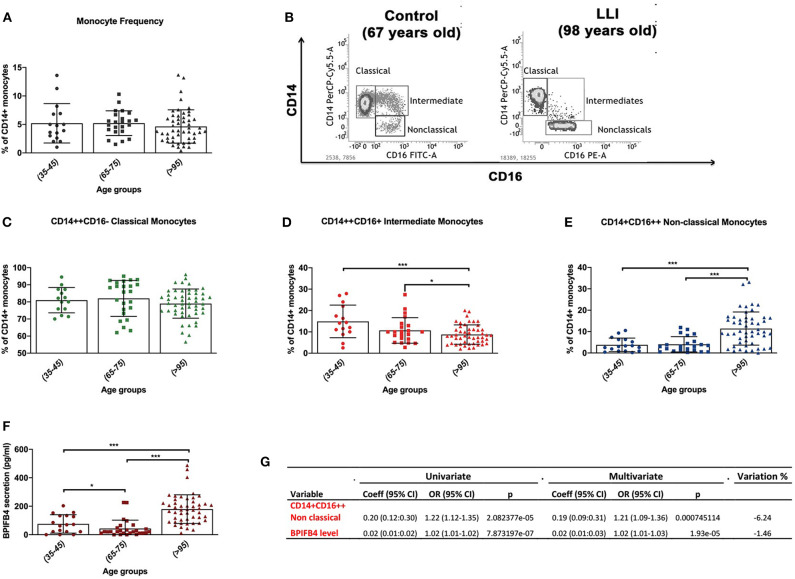
Characterization of monocytic dynamics in long living individuals (LLIs). **(A)** Monocytes frequency in LLIs (median age 97, range 95–99, *N* = 52) expressed by percentage of total CD14+ positive cells using cytofluorimetric analysis. Control group is subdivided in adults (35–45 years, *n* = 18) and old volunteers (65–75 years, *n* = 24). **(B)** Representative FACS gates displaying the relative abundance of different monocyte cell subsets based on the expression of CD14+ and CD16+ markers among freshly isolated PBMC from control volunteer (*left plot, 67 years-old male*) vs. LLI (*right plot, 98 years-old male*). **(C–E)** Relative abundance of CD14++CD16– classical monocytes (*green scatter plots*), CD14++CD16+ intermediate monocytes (*red scatter plots*) and CD14+CD16++ non-classical monocytes (*deep blue scatter plots*) for the different groups of controls and long living-individuals. **(F)** ELISA quantification of BPIFB4 levels in plasma from control volunteers of different ages (*N* = 52) vs. LLIs (*N* = 52) expressed in mean ± SD. Pairwise comparisons statistically significant are indicated (ANOVA; *P < 0.05, ***P < 0.001). **(G)** Results from univariate and multivariate logistic regression. Variable, analyzed variable; Coeff (95%), 95% logistic regression coefficient and 95% Confidence Interval; OR (95% CI), Odds Ratio and 95% Confidence Interval; *p, p*-value; Variation %, percentage change between univariate and multivariate coefficients [(multivariate regression coefficient – univariate regression coefficient)/univariate regression coefficient] * 100.

Flow-cytometry results indicate a peculiar distribution of the monocyte pool, which uniquely marks LLIs (Figure 1). Regarding the total circulating monocyte population, we observed no significant variation (*P* < 0.05) in LLIs compared with controls (Figure 1A). Next, subsets of monocytes were considered (Figure 1B): CD14++CD16– *classical monocytes*, CD14++CD16+ *intermediate monocytes* and CD14+CD16++ *non-classical monocytes* (Supplementary Figure 1). Interestingly, classical monocytes did not differ between groups (Figure 1C), whereas intermediate CD14++CD16+ monocytes were reduced (Figure 1D, *P* < 0.05) and non-classical CD14+CD16++ monocytes were significantly increased in LLIs compared to young and old controls (Figure 1E, *P* < 0.001). Next we confirmed LLIs have higher levels of BPIFB4 compared with both young (35–45 years) and normally aged (65–75 years) control groups, pointing to BPIFB4 as a *bona fide* biomarker of exceptional longevity (Figure 1F). To this end, univariate and multivariate logistic regression was applied to evaluate the association of the variables “non-classical CD14+CD16++ monocytes” and “BPIFB4 level” on the longevity phenotype using data from 97 subjects. As reported in Figure 1G the two variables are independently associated with longevity, both increasing significantly the probability of being long living individuals when included in a multivariate model (Odds Ratio > 1, *p* < 0.001). Further, the percentage variation between regression coefficients from univariate and multivariate logistic regression was −6.24% for non-classical CD14+CD16++ monocytes while −1.46% for BPIFB4 level, thus both lower than the suggested threshold corresponding to 10% commonly used to identify confounders (10).

The enriched subset of non-classical monocytes is known to actively *patrol* the vasculature and remove damaged cells in several disease conditions, thereby aiding tissue healing and the resolution of inflammation (11). Recent intravital imaging has been crucial to definitively elucidate the molecular mechanisms and migratory phenotype of patrolling as preeminent vascular housekeepers (12, 13). The concept of “patrolling monocytes” (PMo) originally referred to mouse (Ly6C^low^) rather than human cells (CD14+CD16++). However, late evidences that differential expression patterns of certain molecules between the 2 major subsets (classical and non-classical monocytes) are shared in humans and mice, have contributed to strengthening the proposed homology and the functional similarities between species (14–16).

Circulating levels of PMo often reflect their infiltration within the parenchyma of several tissues in most of age-related diseases, including cancer, cardiovascular diseases, stroke, neurological damage, arthritis. In myocardial infarction, patrolling monocytes have been associated with reparative, proangiogenic, and pro-arteriogenic effects (17–19). Moreover, their activity in the clearance of amyloid beta from the brain vasculature may suggest a protective action also in neurodegeneration (20).

To date, limited and conflicting data from mice (21) and humans (22) indicated that monocyte subsets may change during aging. Even if the associative nature of data does not permit to conclude the skewed monocyte profile is relevant to the prolonged health-span of the studied LLIs, our present work constitutes the first study to describe a predominant monocyte subset in people that reach extreme ages (>95 years).

Indeed an age-related trend for M2 subsets of circulating monocytes has been partially addressed by Costantini et al. (23). They showed that the healthy aging (>65 years) is associated without significant changes in the frequency of the three monocyte subsets. This is in agreement with our controls' stratification whose analysis highlighted a significant increase of non-classical monocytes frequency only if one compares both younger (35–45 years) or older controls (65–75 years) with LLIs population (>95 years). Indeed, according to Costantini, no significant differences in patrolling frequency were reported in older controls (65–75 years) compared to younger ones (35–45 years). Additionally, Costantini et al. also highlighted that healthy aging is associated with an increase in CD163+ non-classical monocytes while acute myocardial infarct (AMI) patients are characterized by a greater frequency of non-classical CD80 M1 cells. This result even though supports the importance in disease prevention of pro-resolving and anti-inflammatory phenotype of monocytes, left unexplored the functional significance of age-related monocyte phenotype changes in terms of macrophage differentiation, that here we set out to better underpin.

We now know that, in response to an inflammatory trigger, macrophage differentiation from circulating monocytes occurs in tissues in concomitance with the acquisition of a functional phenotype depending on the local environment and classified according to their function (24).

Accumulating evidence indicates non-classical patrolling monocytes might serve as the major precursor for tissue resident macrophages or as precursors for alternatively activated macrophages during inflammation (25–28). Indeed non-classical monocytes have been seen to differentiate into protective M2-macrophages during soft tissue injury (25). Furthermore, in a murine model of rheumatoid arthritis non-classical monocytes firstly differentiate into inflammatory M1-like macrophages and then these cells polarize toward the M2-anti-inflammatory phenotype (26). Accordingly, it makes sense that the deficiency of NR4A1, the transcription factor that non-classical monocytes depend upon for maturation, causes hyper-inflammatory M1-lesional macrophages, leading to worsened atherosclerotic plaques (27, 28).

**Figure 2:**
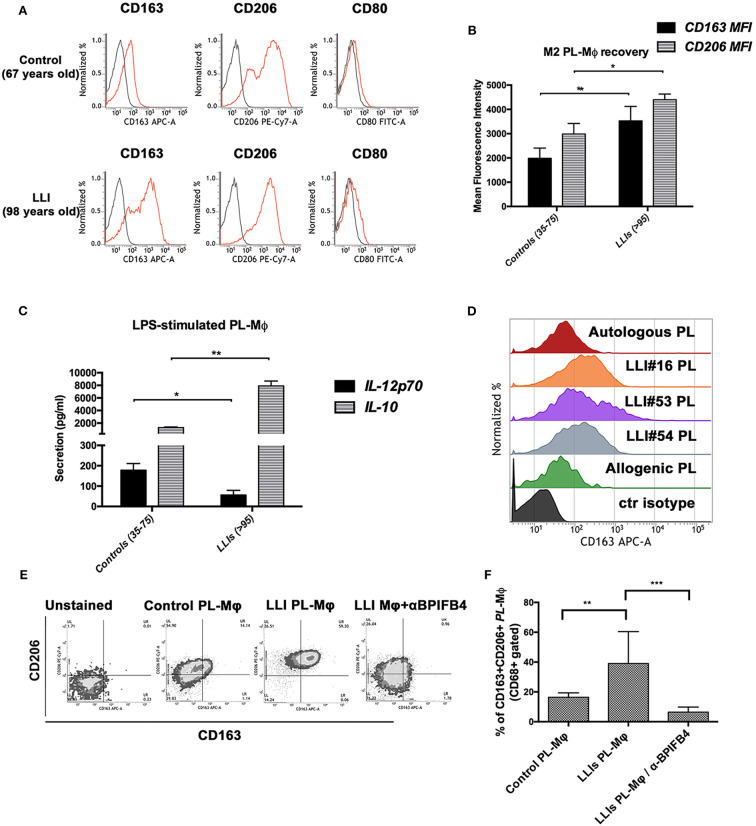
*In vitro* conditioning with plasma from long living individuals leads to polarization of LLIs and controls macrophages toward M2 phenotypes. Macrophages were generated from peripheral blood monocytes of controls (35–75 years, *N* = 10) and of long living individuals (range 95–99, *N* = 10) upon 7 days *in vitro* culture with 20% autologous plasma. **(A)** Panel shows FACS histogram profiles of CD206, CD163, and CD80 protein levels at the cell surface of recovered MPL-macrophages (viable gated CD68+ cells) from a representative control volunteer (*upper plots, 67 years-old male*) vs. a representative LLI (*lower plots, 98 years-old male*). Cell staining was gated using isotype control antibodies (*gray histograms*). **(B)** Bars graph in panel report the mean fluorescence intensity (MFI) values ± SD of CD163 and CD206 M2 marker on viable CD68+ gated cells from controls (*N* = 10) and LLIs (*N* = 10). Pairwise comparisons statistically significant are indicated (ANOVA; *P < 0.05 and **P < 0.01). **(C)** IL-12p70 and IL-10 secretion by control and LLIs macrophages after 1 μg/ml LPS stimulation for 24 h. Cell culture supernatants were collected and cytokines secretion was determined using bead-based multiplex ELISA. Results were expressed as the mean ± SD of all sample determinations conducted in duplicate. All pairwise comparisons are statistically significant (ANOVA; *P < 0.05, **P < 0.01). **(D–F)** Peripheral blood monocytes of control volunteers (35–75 years, *N* = 10) were 7 days-exposed to plasma from LLIs (*N* = 10) and autologous or allogenic control plasma as comparison, in the presence or absence of BPIFB4 (1:100) blocking antibodies for the last 72 h of culture. After 7 days *in vitro* culture, cytofluorimetric analysis of recovered MPL-macrophages was conducted. **(D)** FACS histogram profiles for CD163 M2 markers in MPL-macrophages of both control plasma- and 3 representative LLIs-treated cells are shown. **(E)** Representative flow cytometry CD206 vs. CD163 density plots for each experimental condition is presented. **(F)** Bars graph report the percentage ± SD of CD206+CD163+ of gated MPL-macrophages from ten independent experiments using different donors (ANOVA; **P < 0.01, ***P < 0.001).

We sought therefore to examine whether the LLIs' plasma could shift the phenotype of monocyte-derived macrophages toward the pro-resolving M2 (alternatively activated) or pro-inflammatory M1 phenotype. To this end, CD14+ monocytes purified from blood of LLIs (range 95–99, *N* = 10) or controls (35–75 years) were conditioned with autologous plasma (added to serum-free base medium) and induced to differentiate *ex vivo* into macrophages. As reported in Figure 2A, control macrophages harvested at the end of the conditioning period manifested an M1-M2 intermediate profile displaying the canonical CD206+/CD163–/CD80^low^ phenotype. On the contrary, LLIs' macrophages showed an enriched M2 phenotype as highlighted by higher surface level of both CD206 and of the anti-inflammatory marker CD163 (Figures 2A,B). Moreover, macrophages from LLIs secreted higher anti- (IL-10) and lower pro-inflammatory (IL-12p70) cytokines compared to control macrophages (Figure 2C).

Next, we asked if the favorable profile of the LLIs plasma could be transferred in an allogenic setting, too. Monocytes from control subjects showed a huge increase of the percentage of CD163++ macrophages (M2 polarizing effect) when treated with the LLIs' plasma (Figure 2D). On the other hand, the plasma from controls did not influence autologous and heterologous monocytes (Figure 2D). To be noted, donor's plasma did not interfere with LLI's monocytes phenotype (Supplementary Figure 2), suggesting that not only the functional characteristics of LLI's monocytes but rather the presence of peculiar soluble factors in LLIs' plasma may be primarily responsible for M2 skewing.

Knock-down of *BPIFB4* or mutagenesis of protein at the functional serine 75 residue is known to abolish the BPIFB4 capacity to activate eNOS signaling and sustain endothelial function (9). Here, we used a blocking antibody, designed on the BPIFB4 serine 75 phosphorylation site, to verify whether, among different soluble cytokines, BPIFB4 is responsible for the ability of the LLIs' plasma to promote M2 polarization of monocytes from control subjects. Interestingly, the BPIFB4-neutralizing antibody induced a significant decrease in macrophage M2 (CD206+/CD163+) recovery (Figures 2E,F) upon stimulation with LLIs' plasma.

M2 macrophages have a role in protecting from many diseases associated with aging. Hence, a plausible scenario emerging from our study is that high levels of circulating BPIFB4 favor M2 polarization thereby creating an innate immunity landscape supportive to disease resistance and moderation of inflammatory reactions (see cartoon). The fact that the skewing property of the LLIs' plasma is transferrable to controls but not *vice versa* (Supplementary Figure 2) suggests the presence of factors, including high levels of BPIFB4, endowed of immunomodulatory therapeutic potential. Further *in vivo* titration studies are necessary to determine the effective dosage and ability of BPIFB4 to support the M2 skewing under acute and chronic inflammatory conditions.

## Materials and Methods

### Patients Recruitment

The study was performed on a group of 104 individuals: *n* = 52 control volunteers (median age 55, range 35–75) and *n* = 52 long-living individuals LLIs (median age 97, range 95–99). Control volunteers group was divided in middle-aged (35–45 years) and old(er) group (65–75 years). These were constituted, respectively, by healthy donors and by subjects with no apparent diseases who underwent routine preventive laboratory tests. For each, venous blood (10 mL) was withdrawn for analyses and detailed anamnesis was collected. For detailed information concerning LLIs group see Table 1. All participants signed an informed consent for the management of personal anamnestic data and blood samples. The study was approved by the IRCCS MultiMedica ethical committee and conducted in accordance with the ethical principles deriving from the Declaration of Helsinki.

**Table 1:** Clinical Characteristic of LLIs' group.

**#Subject**	**Gender**	**Age (years)**	**Major desease**	**Drug treatments**
L01	F	98	HT, DM	ARBs, ASA, βB, Gl, Metformin
L02	M	95	HT, OP	ARBs ASA, ST
L03	M	95	COPD, HHD	ASA, ST, Tiotropium Br
L04	F	98	DM, HHD, OP, CV	ASA, LD, Gl, Gl
L05	F	95	HT, OP, CV	ASA, ST, ACEis
L06	M	95	HT, COPD	ST, ACEis, CCB, Tiotropium Br
L07	F	95	HT, DM, OP, CV	ASA, βB, Gl
L08	F	95	HT, CV	ACEis
L09	M	96	HT	CCB, ACEis
L10	F	96	HT, OA	Indapamide
L11	F	98	HT, CV	ARBs, ASA, ST, βB
L12	M	95	CV	ASA
L13	M	96	COPD, HT, HF	ARBs, ASA, βB, LD, Tiotropium Br
L14	M	96	HT, DM, CV	ARBs, ST, Gl, Metformin
L15	M	95	HT, HHD, CV	LD, CCB
L16	F	98	HT, DM	ARBs
L17	F	99	OA	Nimesulide
L18	F	95	COPD, HHD	CCB, Tiotropium Br
L19	F	96	HT, DM	ARBs, Gl, Metformin
L20	F	100	HT	ACEis
L21	F	97	HHD	ARBs
L22	F	95	HT	ASA
L23	F	96	HF	Spironolactone, Nitroglycerin
L24	M	96	HT	LD
L25	F	95	HT	βB
L26	F	99	DM, HHD	LD, Metformin
L27	F	97	HT, HHD	LD
L28	F	96	HHD	ASA
L29	F	96	HT, HF	Digoxin
L30	F	95	HT	ASA, βB
L31	F	95	HT, DM	ASA, βB, Metformin
L32	M	98	/	/
L33	F	96	HT, COPD	ASA, LD, Tiotropium Br
L34	F	95	/	/
L35	F	95	HT	CCB, ACEis
L36	F	98	DM	Gl
L37	F	97	HT	CCB
L38	F	96	HT	ARBs, Indapamide
L39	F	95	HT, OP, CV	ARBs
L40	M	96	HT, CV	ACEis
L41	F	103	HT, DM, OP	LD, Metformin
L42	F	95	DM, HHD	LD, Gl
L43	F	98	OP, CV	ACEis
L44	M	95	HHD	CCB
L45	F	97	HT	ACEis
L46	M	95	HHD, HF	βB, LD
L47	F	97	CV	Lorazepam
L48	F	98	HT, CV, DM	CCB, Metformin, ACEis
L49	F	98	HT, DM	ARBs, ASA
L50	M	97	HT	ACEis
L51	M	96	HT, HF	ASA, βB
L52	F	99	HT	ST

*Major disease: HT, hypertension; DM, diabetes mellitus; COPD, chronic obstructive pulmonary disease; HHD, hypertensive heart disease; OP, osteoporosis; CV, cerebral vasculitis; OA, osteoarthritis; HF, heart failure*.

*Drug treatments: ARBs, angiotensin II receptor blockers; ST, statin; ASA, acetylsalicylic acid; βB, β beta blockers; LD, loop diuretics; ACEis, angiotensin-converting-enzyme inhibitors; CCB, calcium channel blockers; Gl, glinides*.

#### CD14+ Monocytes Isolation and Macrophage Generation

Peripheral Blood Mononuclear Cells (PBMC) were extracted from whole blood by density gradient (Ficoll). After separation, PBMC were collected and washed for the subsequent experiments.

CD14+ monocytes were positively selected from PBMCs by an immunomagnetic procedure (Miltenyi Biotec). Then, CD14+ cells were induced to differentiate in M1 or M2 macrophages using reagents included in the CellXVivo™ Human M1 or M2 Macrophage Differentiation Kit (R&D system), or alternatively in the presence of 20% human plasma (MPL). Where indicated, plasma-stimulated monocytes were cultured in serum free base media (R&D system) with or without BPIFB4 blocking antibody. Blocking antibody for BPIFB4 was purchased from CliniSciences S.r.l.-Guidonia Montecelio—Italy.

All cell cultures were conducted at 37C° in humidified 5% CO_2_ atmosphere.

#### Antibodies and Flow Cytometry

Single-cell suspensions were stained with mAb against human CD14 (HCD14; BioLegend; 1:20), CD16 (REA423; Miltenyi Biotec GmbH; 1:50), CD206 (DCN228; Miltenyi Biotec GmbH; 1:11), CD163 (REA812; Miltenyi Biotec GmbH; 1:50), CD68 (Y1/82A; BioLegend; 1:20), CD80 (2D10; BioLegend; 1:20).

After 20 min incubation at 4°C in the dark, cells were washed and resuspended in PBS for the FACS analysis. For each test, cells was analyzed using FACS Verse Flow Cytometer (BD Biosciences).

#### Cytokines Detection

Beads-based multiplex ELISA (LEGENDplex, Biolegend, USA) was used to measure cytokines in macrophage supernatants. Diluted cell culture supernatants were incubated for 2 h with the beads and detection antibodies, followed by 30 min incubation with SA-PE. After washing, beads were resuspended in washing buffer and acquired using a FACS VERSE flow cytometer (BD Biosciences). Data were analyzed with the LEGENDplex Data Analysis Software.

Plasma levels of BPIFB4 were measured using ELISA Kit (Cusabio CSB-YP003694HU) following the manufacturer's protocol.

### Statistical Analysis

In all other experiments shown, statistical analysis was performed by using the GraphPad prism 6.0 software for Windows (GraphPad software). For each type of assay or phenotypic analysis, data obtained from multiple experiments are calculated as mean ± SD and analyzed for statistical significance using ANOVA for multiple comparison *p* < 0.05 were considered significant. ^*^p < 0.05, ^**^p < 0.01, and ^***^p < 0.001. Logistic regression analyses were performed by the R software tool (www.r-project.org).

## Data Availability Statement

The datasets generated for this study are available on request to the corresponding author.

## Ethics Statement

This study was approved by the IRCCS MultiMedica ethical committee and conducted in accordance with the ethical principles deriving from the Declaration of Helsinki. The patients/participants provided their written informed consent to participate in this study.

## Author Contributions

EC designed and conducted the study. FM, PS, VL, AF, and MC performed laboratory activities. AC, CV, and PM cared for the subjects of the study, evaluation of their health status, and reviewed critically the paper. AM performed statistical analysis. AP and EC performed statistical analysis, data interpretation, and wrote the manuscript. AP coordinated the research team and provided financial support. All authors approved the final version to be published.

## Conflict of Interest

The authors declare that the research was conducted in the absence of any commercial or financial relationships that could be construed as a potential conflict of interest.

## Supplementary Material

Supplementary Figure 1 Gating Strategy of the three monocyte subsets based on relative CD14 and CD16 expression. Flow cytometry dot plot showing the gating of classical, intermediate, and non-classical monocyte subsets. From the forward/side scatter plot monocytes were first selected. Then as by definition the intermediate and classical monocyte subsets possess the same levels of CD14, we found it convenient to use the end point of CD14 expression by the classical monocytes as a set point to segregate between the intermediate and non-classical subsets.

Supplementary Figure 2 *In vitro* conditioning of LLIs' monocytes with plasma PL from controls. Peripheral blood monocytes of LLIs (>95 years, *N* = 3) were 7 days-exposed to allogenic plasma PL from control volunteers (35–75 years, *N* = 3) to test the inner ability of LLIs monocytes to acquire an M2 phenotype. After 7 days *in vitro* culture, cytofluorimetric analysis of recovered MPL-macrophages was conducted. Bars graph report both the Mean Fluorescence Intensity and the percentage ± SD of CD163+ (M2 marker) of gated MPL-macrophages from three independent experiments using different donors. The skewing effects of LLIs's plasma (Autologous LLIs' PL) on LLIs's cells and autologous control plasma (Autologous PL) on monocytes of their own (control monocytes) are also shown for comparison (ANOVA; ^*^*P* < 0.05).
