# Supplementary figures and images for: Circulating BPIFB4 Levels Associate With and Influence the Abundance of Reparative Monocytes and Macrophages in Long Living Individuals
Source: Front Immunol. 2020 May 29;11:1034. doi: 10.3389/fimmu.2020.01034 (PMC7272600; doi:10.3389/fimmu.2020.01034)

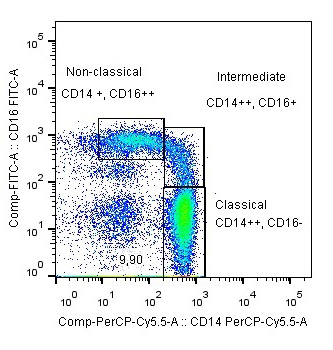

Supplement: Supplementary Figure 1 — Gating Strategy of the three monocyte subsets based on relative CD14 and CD16 expression. Flow cytometry dot plot showing the gating of classical, intermediate, and non-classical monocyte subsets. From the forward/side scatter plot monocytes were first selected. Then as by definition the intermediate and classical monocyte subsets possess the same levels of CD14, we found it convenient to use the end point of CD14 expression by the classical monocytes as a set point to segregate between the intermediate and non-classical subsets. [file Image_1.jpg]

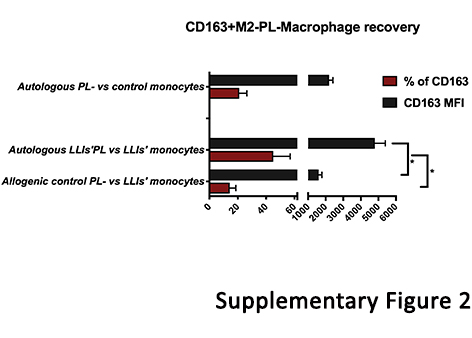

Supplement: Supplementary Figure 2 — In vitro conditioning of LLIs' monocytes with plasma PL from controls. Peripheral blood monocytes of LLIs (>95 years, N = 3) were 7 days-exposed to allogenic plasma PL from control volunteers (35–75 years, N = 3) to test the inner ability of LLIs monocytes to acquire an M2 phenotype. After 7 days in vitro culture, cytofluorimetric analysis of recovered MPL-macrophages was conducted. Bars graph report both the Mean Fluorescence Intensity and the percentage ± SD of CD163+ (M2 marker) of gated MPL-macrophages from three independent experiments using different donors. The skewing effects of LLIs's plasma (Autologous LLIs' PL) on LLIs's cells and autologous control plasma (Autologous PL) on monocytes of their own (control monocytes) are also shown for comparison (ANOVA; *P < 0.05). [file Image_2.jpg]
